# Melatonin imparts tolerance to combined drought and high-temperature stresses in tomato through osmotic adjustment and ABA accumulation

**DOI:** 10.3389/fpls.2024.1382914

**Published:** 2024-03-28

**Authors:** Annadurai K. Mumithrakamatchi, Senthil Alagarswamy, Kuppusamy Anitha, Maduraimuthu Djanaguiraman, M. Karuppasami Kalarani, Ramakrishnan Swarnapriya, Subramanian Marimuthu, Sampathrajan Vellaikumar, Selvaraju Kanagarajan

**Affiliations:** ^1^ Department of Crop Physiology, Tamil Nadu Agricultural University (TNAU), Coimbatore, India; ^2^ Directorate of Crop Management, Tamil Nadu Agricultural University, Coimbatore, India; ^3^ Floriculture Research Station, Thovalai, India; ^4^ Department of Agronomy, Agricultural College and Research Institute (AC&RI), Eachangkottai, Thanjavur, India; ^5^ Centre for Plant Molecular Biology and Biotechnology, Tamil Nadu Agricultural University, Coimbatore, India; ^6^ Department of Plant Breeding, Swedish University of Agricultural Sciences, Lomma, Sweden; ^7^ School of Science and Technology, The Life Science Centre, Örebro University, Örebro, Sweden

**Keywords:** melatonin, drought, high-temperature, stomata, proline, abscisic acid, trichomes, water use efficiency

## Abstract

In recent years, environmental stresses viz., drought and high-temperature negatively impacts the tomato growth, yield and quality. The effects of combined drought and high-temperature (HT) stresses during the flowering stage were investigated. The main objective was to assess the effects of foliar spray of melatonin under both individual and combined drought and HT stresses at the flowering stage. Drought stress was imposed by withholding irrigation, whereas HT stress was imposed by exposing the plants to an ambient temperature (AT)+5°C temperature. The drought+HT stress was imposed by exposing the plants to drought first, followed by exposure to AT+5°C temperature. The duration of individual and combined drought or HT stress was 10 days. The results showed that drought+HT stress had a significant negative effect compared with individual drought or HT stress alone. However, spraying 100 µM melatonin on the plants challenged with individual or combined drought and HT stress showed a significant increase in total chlorophyll content [drought: 16%, HT: 14%, and drought+HT: 11%], F_v_/F_m_ [drought: 16%, HT: 15%, and drought+HT: 13%], relative water content [drought: 10%, HT: 2%, and drought+HT: 8%], and proline [drought: 26%, HT: 17%, and drought+HT: 14%] compared with their respective stress control. Additionally, melatonin positively influenced the stomatal and trichome characteristics compared with stress control plants. Also, the osmotic adjustment was found to be significantly increased in the melatonin-sprayed plants, which, in turn, resulted in an increased number of fruits, fruit set percentage, and fruit yield. Moreover, melatonin spray also enhanced the quality of fruits through increased lycopene content, carotenoid content, titratable acidity, and ascorbic acid content, compared with the stress control. Overall, this study highlights the usefulness of melatonin in effectively mitigating the negative effects of drought, HT, and drought+HT stress, thus leading to an increased drought and HT stress tolerance in tomato.

## Introduction

Climate change introduces multiple abiotic stresses that have a widespread impact throughout the crop life cycle. This leads to a significant reduction in the productivity of horticultural crops by approximately 50% to 70% (Boyer, 1982; Francini and Sebastiani, 2019). Recent research confirmed that developed countries are more susceptible to climate change, showing a global susceptibility index ranging from 8% to 11%, posing a serious threat to global food security (Lesk et al., 2016). Among these stresses, drought and high-temperature (HT) are more prevalent, adversely affecting crop growth and productivity (Zandalinas et al., 2018). Drought occurs due to an imbalance between the evapotranspiration flux (water loss through evaporation and plant transpiration) and the intake of water from the soil (Lipiec et al., 2013). On the other hand, HT stress depends on an increase in air temperature beyond a specific threshold level for a particular time, which ultimately harms plant growth and development (Zhao et al., 2017). Toward the end of twenty-first century, there exists a greater probability of drought occurrence due to an estimated increase in 2.8°C temperature (Singh et al., 2022). Many findings suggested that the effect of drought and HT stress in an association is more detrimental than individual stresses (Kumari et al., 2018; Zhou et al., 2019; Liu et al., 2020; Hosseini et al., 2021). The frequent occurrence of drought and temperature variations negatively affects crop adaptability, therefore necessitating the improvement of stress tolerance ability to enhance the crop yield to meet the increasing food demands of a growing population (Kang et al., 2009).

Tomato, a widely consumed vegetable crop, holds global significance and originated from South America (Bai and Lindhout, 2007). Tomato is rich in carotenoids, especially lycopene, and offers potential benefits in preventing cancer, reducing cardiovascular risk, and delaying cellular ageing (Di Cesare et al., 2012). In India, tomato cultivation covers an area of 8.41 lakh ha, with an annual production of 20 million metric tons in 2020–2021 (Indiastat, 2022). Previous research has confirmed that when drought and HT stress occur together, it has a more detrimental effect on canopy temperature, chlorophyll content, and relative leaf water content (Sairam et al., 1997; Shah and Paulsen, 2003; Zhou et al., 2017; Thakur et al., 2022). This impact is attributed to the crucial role in sustaining the photosynthetic process, and the production of carbon assimilates. According to Aprile et al. (2013), changes in canopy temperature play a significant role in assessing a plant’s ability to regulate water loss and prevent overheating, thus contributing to maintaining higher water use efficiency (WUE). The non-destructive method of chlorophyll fluorescence is observed to exhibit a greater decrease under combined stress compared with individual drought or HT stress, aiding in the evaluation of photosynthetic performance (Netshimbupfe et al., 2022). Additionally, proline, an osmoregulant, assists in maintaining osmotic adjustment by reducing the osmotic potential and sustaining turgor pressure when plants are under stress (Al-Yasi et al., 2020). Also, abscisic acid, an important hormone, plays a key role in stomatal regulation. Drought stimulates ABA production and causes stomatal closure to reduce transpiration and water loss, whereas HT prompts stomatal opening to induce leaf cooling (Chaves et al., 2016), ultimately affecting the fruit yield and quality (Jiang et al., 2020; Venancio et al., 2020). On the other hand, the number of trichomes determines water use efficiency in tomato, thus affirming their ability to withstand stress conditions (Galdon-Armero et al., 2018). Therefore, the negative effects of drought and HT stress could potentially be mitigated by the use of phytohormones, which can be an effective management approach (Yadav et al., 2021).

Plants themselves possess a mechanism to perceive signals, communicate, and respond to various abiotic stress factors, which are mediated by phytohormones (Davies, 2010). A pleiotropic molecule, melatonin, has a significant role in mitigating the detrimental effects of abiotic stress, involving growth and development (Liang et al., 2019), seed germination (Castanares and Bouzo, 2019), root architecture (Altaf et al., 2022), photosynthesis and antioxidant defense (Anitha et al., 2023), stomatal regulation (Jensen et al., 2023), and fruit yield and quality (Dou et al., 2022). Several findings have investigated the effect of melatonin on physiological and biochemical mechanisms under individual drought or HT stresses (Ibrahim et al., 2020; Jahan et al., 2021). Despite many studies, researchers’ recent interest has emerged in studying the effect of melatonin under drought and HT stress in combination.

In this specific context, an interesting aspect highlighting melatonin’s potential as an antioxidant defense mechanism against ROS-induced oxidative damage under individual and combined drought and HT stress in tomato has been studied in detail (Zhang et al., 2014; Annadurai et al., 2023). Despite several findings, there is no evidence evaluating the performance of melatonin on osmotic regulation, stomatal regulation, trichome morphology, and water use efficiency. Our study aims to investigate the effect of melatonin under drought, HT, and combined stress. Therefore, objectives are framed to (i) evaluate the response of canopy temperature, chlorophyll content, photosystem II efficiency (F_v_/F_m_), and relative water content under different stress conditions, (ii) investigate the mechanism involved in the osmotic and stomatal regulation, (iii) assess trichome characteristics and instantaneous water use efficiency (WUE), and (iv) observe the effect of melatonin on the fruit yield and quality. This study hypothesized that foliar spray of melatonin could help in alleviating the negative effects of combined drought and HT stress by reducing canopy temperature, preventing chlorophyll degradation, maintaining osmotic balance, preventing excessive water loss, and improving water use efficiency, which could be the reason for increased fruit yield, and quality improvement in tomato.

## Materials and methods

### Site characteristics and experimental material

The pot culture experiment was conducted from March to June 2022 in the Glasshouse and Open Top Chamber (OTC) at the Department of Crop Physiology, Tamil Nadu Agricultural University, Coimbatore, India. The tomato hybrid ‘Shivam’ is of semi-determinate type with a total crop duration of 130 to 135 days, and it was cultivated in portrays filled with a mixture of vermicompost and coir pith in a ratio of 3:1. Until 21 days, the seedlings were maintained under controlled conditions at ambient temperature. Later, the seedlings were transplanted in a large-sized plastic pot of 46 cm in length and 60 cm in diameter. The pot mixture comprised a mixture of red soil, sand, and vermicompost in a ratio of 3:1:1. To each pot, urea (4.2 g), single superphosphate (21.4 g), muriate of potash (8.5 g), borax (0.85 g), zinc sulfate (4.2 g), and copper sulfate (0.32 g) was added as basal. Life irrigation was provided on the third day after transplanting (DAT), and subsequent irrigation was done on alternate days. Gap filling was carried out on the 7^th^ DAT. Neem oil (1%) was sprayed to control leaf miner infestation during the early vegetative stage, and subsequently, yellow sticky traps were placed to control sucking pests. Top dressing of urea (12 g) and muriate of potash fertilizer (12 g) was done at 30 days intervals until 90 days from planting. The tomato plants were supported with a stake at the 35^th^ DAT (Crop Production Guide of Horticulture Crops Directorate of Horticulture andPlantation Crops and TNAU, 2020).

### Treatment details and stress imposition

A completely randomized block design experiment was conducted with two factors and four replications. The first factor consists of three levels representing different types of stress: drought, high-temperature (HT), and combined drought and high-temperature (Drought+HT). The second factor had four levels, representing different foliar treatments: absolute control (AC), stress control (SC), 80 µM melatonin (80 µM Mel), and 100 µM melatonin (100 µM Mel). The plants were initially kept under well-watered conditions and at ambient temperature until the flower initiation stage. Upon reaching flowering, the plants were exposed to different stress conditions for a duration of 10 days. The plants experiencing drought stress were maintained in a Glasshouse, whereas those subjected to HT stress and combined drought + HT stress were maintained in an Open Top Chamber. The 12 experimental treatment combinations containing the details of stress imposition and time of foliar spray are mentioned in **Table 1**.

**Table 1:** Details of stress imposition, and time of foliar spray during the experimental period.

Stress	Foliar spray	Stress imposition and time of foliar spray
Drought	Absolute control (AC)	Ambient temperature, maintained at 100% field capacity, received no spray
	Stress control (SC)	Ambient temperature, withholding irrigation, received no spray
	80 µM melatonin (80 µM Mel)	Ambient temperature, withholding irrigation, received spray on the 4^th^ day after onset of stress
	100 µM melatonin (100 µM Mel)	Ambient temperature, withholding irrigation, received spray on the 4^th^ day after onset of stress
HT	Absolute control (AC)	Ambient temperature, maintained at 100% field capacity, received no spray
	Stress control (SC)	Ambient temperature (AT) +5°C, maintained at 100% field capacity, received no spray
	80 µM melatonin (80 µM Mel)	Ambient temperature (AT) +5°C, maintained at 100% field capacity, received spray on the 4^th^ day after onset of stress
	100 µM melatonin (100 µM Mel)	Ambient temperature (AT) +5°C, maintained at 100% field capacity, received spray on the 4^th^ day after onset of stress
Drought+HT	Absolute control (AC)	Ambient temperature, maintained at 100% field capacity, received no spray
	Stress control (SC)	Withholding irrigation followed by ambient temperature (AT) +5°C, received no spray
	80 µM melatonin (80 µM Mel)	Withholding irrigation followed by ambient temperature (AT) +5°C, received spray on the 4^th^ day after onset of stress
	100 µM melatonin (100 µM Mel)	Withholding irrigation followed by ambient temperature (AT) +5°C, received spray on the 4^th^ day after onset of stress

### Soil moisture and weather data

The stress duration is determined by the reduction in soil moisture observed through the theta probe, which was calculated based on a decrease from 100% field capacity during drought conditions. In HT-stressed plants, the duration depends on the decrease in relative humidity percentage. In case of drought + HT stress, the duration of stress is determined by the reduction in soil moisture percentage along with the decrease in relative humidity percentage. Throughout the period of stress imposition, the relative humidity ranged from 47% to 75%. The figures representing the soil moisture data and meteorological data for the open-top chamber (OTC) align with the findings of the previous article published by Annadurai et al. (2023).

### Canopy temperature and chlorophyll fluorescence

A portable infrared thermometer (FLIR E6, OR, USA) was used to measure the canopy temperature, placed inclined at an angle of 45°, and the observation was recorded at a distance of 1 m from the top canopy cover (Silva et al., 2018). The third fully expanded leaf below the apex was used to measure the chlorophyll fluorescence using a chlorophyll fluorometer (Dalal and Tripathy, 2018). The middle portion of the leaf without veins was dark adapted for 30 min using leaf clips. The maximal photochemical efficiency of PSII (F_v_/F_m_) was recorded. The readings of canopy temperature and chlorophyll fluorescence were taken between 10:30 am to 12:30 pm.

### Photosynthetic pigments

The chlorophyll content was measured in the leaves obtained from the uppermost third branch on the 10^th^ day after the onset of stress. The photosynthetic pigments were determined using the acetone method as described by Arnon (1949). The required quantity of leaf sample, excluding veins, was macerated with 10 mL of 80% acetone and then centrifuged at 3,000 rpm for 15 min. The collected supernatant is made to a final volume of 25 mL using 80% acetone. The absorbance readings were taken at 645 nm and 663 nm to quantify chlorophyll a, chlorophyll b, and total chlorophyll using the following formula:


$$Chlorophyll\;a\;\left(mg\;g^{-1}\;FW\right)=12.7\left(A_{663}\right)-2.69\left(A_{645}\right)\times \frac{V}{1000\times W}$$

$$Chlorophyll\;b\;\left(mg\;g^{-1}\;FW\right)=22.9\left(A_{645}\right)-4.68\;\left(A_{663}\right)\times \frac{V}{1000\times W}$$

$$Total\;chlorophyll\;\left(mg\;g^{-1}\;FW\right)=Chlorophyll\;a+Chlorophyll\;b$$

where V is the final volume of extract and W is the leaf fresh weight in grams.

### Relative water content

The fresh leaves obtained from the uppermost second branch were punched into 50 bits of equal size, and their fresh weight (FW) was recorded. Subsequently, the leaf bits were immersed in distilled water, and after 24 h, the turgid weight (TW) was noted by blotting with tissue paper to remove surface moisture, following the procedure as mentioned in Netshimbupfe et al. (2022). Next, the leaf samples were placed in a hot air oven at 60°C for 48 h, and the dry weight (DW) was recorded. The leaf relative water content (RWC) was calculated according to Barrs and Weatherley (1962) using the formula:


$$Relative\;water\;content\;\left(\%\right)=\frac{Fresh\;weight\;\left(FW\right)-Dry\;weight\;\left(DW\right)}{Turgid\;weight\;\left(TW\right)-Dry\;weight\left(DW\right)}\times 100$$

### Osmotic adjustment

The osmotic potential of the leaf was determined using a vapor pressure osmometer (Vapor Model 5520 Wescor Inc., Logan, UT, USA), and the osmometer readings were expressed in mmol kg^−1^. The osmotic potential ( ψ_s_) was expressed in megapascal (MPa) and calculated as per the method of Babu et al. (1999) using the Van’t Hoff equation:


$$Osmotic\;potential\;\left(MPa\right)=\frac{-cRT}{1000}$$

where c is the concentration of sap in mmol kg^−1^, R is the universal gas constant (0.0832 L atm K^−1^ mol^−1^), and T is the laboratory temperature (29°C). The value of osmotic potential is used to calculate the osmotic adjustment according to Wilson et al. (1979), using the following equation:


$$Osmotic\;potential\;\left(\varphi_{S100}\right)=\frac{\varphi_{S}\times RWC}{100}$$

Osmotic adjustment (MPa)=OP (Irrigated)−OP (Stress)

### Proline

Fresh leaves of tomato plants were used to analyze proline content, as described by Bates et al. (1973). A 10 mL volume of 3% sulfosalicylic acid was used to macerate 0.5 g of leaf sample, and then it was centrifuged at 10,000 rpm for 15 min. The mixture consisted of 2 mL of 6 M orthophosphoric acid, 2 mL glacial acetic acid, 2 mL supernatant, and 2 mL ninhydrin reagent, and then contents were kept in a water bath at 80°C for 1 h. Subsequently, the test tubes were allowed to cool at room temperature, and the solution was transferred to the separating funnel. To that solution, 4 mL of toluene was added and shaken for 30 s. The bottom layer was discarded, and the upper layer was used to measure the absorbance at 520 nm. Meanwhile, the standard curve was obtained using proline as a standard, and the proline content was expressed in µmol g^−1^ of fresh weight.

### Stomatal parameters

Stomatal anatomy was assessed in the primary leaflet of the uppermost second branch with four replicates per treatment. The stomatal imprints were obtained from the middle portion of the leaf using the nail polish method (Wu and Zhao, 2017). A thin film of nail polish was applied on the abaxial surface of the leaf in between the veins and allowed to dry for 10 min. Clear tape was used to peel the imprints, and they were fixed on the microscope slide using 80% glycerol, along with a coverslip. The stomatal images were visualized in a Euromex iScope Phase Contrast Microscope, the images were shot directly using an HD Mini color camera (HDMI interface VC.3024), and the measurements of the captured images were obtained from microscope image analysis software (Optscopes). The number of stomata was counted individually by generating 12 images of 0.2 mm^2^ size for each sample in a treatment at 10× magnification and denoted as the number of stomata per mm^2^. The measurement of 15 stomata per replicate was chosen to determine the stomatal length, stomatal width, pore length, and pore width using a line at 40× magnification and expressed in µm units, whereas the stomatal area and pore area were measured using an ellipse, and the readings were expressed in µm^2^.

### ABA content

**Figure 1:**
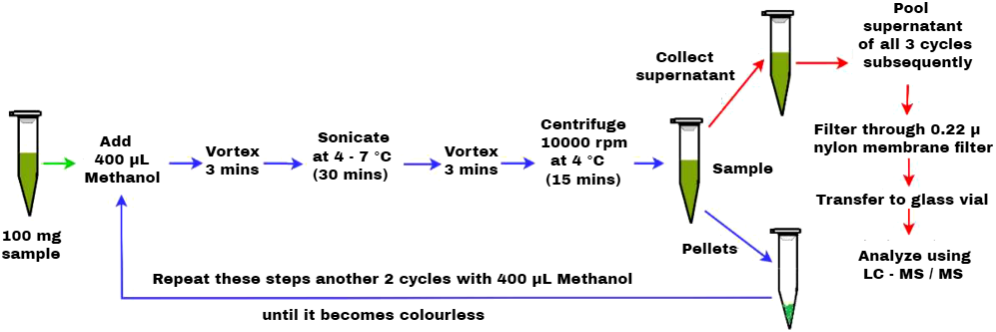
Schematic representation of LC-MS/MS-based extraction protocol for quantification of ABA level.

ABA levels in LC-MS/MS were determined as per the protocol of Muller and Munne-Bosch (2011), with slight modifications. In this modified protocol, methanol was used as a primary solvent at a volume of 400 µL, and the procedure was repeated for three cycles. Fresh samples are excised and frozen immediately in liquid nitrogen. 100 mg of leaf sample was grounded using liquid nitrogen and placed in a 1.5-mL centrifuge tube, to which 400 µL of methanol was added as a solvent and vortexed for 3 min. Then, ultrasonication was performed for 30 min using ice to maintain at 4°C–7°C followed by vortex for 3 min and centrifuged at 10,000 rpm for 15 min at 4°C. The supernatant was collected in a separate centrifuge tube, which was maintained at 4°C, and the procedure was repeated for another two cycles by adding 400 µL of methanol to the pellet until it turned colorless. The supernatant of all three cycles was pooled together and then filtered through 0.22-µ nylon membrane filter. The filtered supernatant was then transferred to a glass vial and stored at − 20°C until analysis (**Figure 1**). The quantification of the ABA level was performed using an N-series LCMS-8045 triple quadrupole liquid chromatograph mass spectrometer (Shimadzu LC-MS/MS) equipped with an electrospray ionization probe (ESI), degassing unit, solvent delivery module, autosampler, column oven, and flow diversion valve. The separation was done using a chromatographic C18 column with the elution of 0.4 mL min^−1^ flow rate for the injection volume of 10 µL, using the gradients of solvent A containing 0.1% formic acid in water and solvent B containing 0.1% formic acid in methanol at different proportions in t (min) as (90, 10, 0), (80, 20, 2), (60, 40, 6), (30, 70,11), (5, 95, 15), (90, 10, 19), and (90, 10, 22). The drying gas flow (10 L min^−1^), heating gas flow (10 L min^−1^), nebulizing gas flow (3 L min^−1^), interface temperature (300°C), dissolvation line temperature (250°C), and heat block temperature (400°C) were fixed to run multiple reaction monitoring (MRM) for a duration of 22 min. The stock solution of 1 ppm concentration was prepared from the serial dilutions made from 1,000 ppm using methanol: water (1:1). The different standard concentrations were used to calibrate the standard curve, which is used for quantification of ABA levels.

### Trichome parameters and water use efficiency

Trichome parameters were measured in a terminal leaflet of fully expanded leaves. The trichome length and width were determined as outlined by Hernandez and Park (2022), similar to stomatal parameters observed at 40× magnification, whereas the number of trichomes was assessed at 10× magnification using a Euromex iScope Phase Contrast Microscope equipped with HD Mini color camera (HDMI interface VC.3024). The trichome length and width were quantified in 15 randomly selected trichomes per replicate using microscope image analysis software (Optscopes) and were determined in µm units. In contrast, the number of trichomes was counted individually by generating 12 images of 0.2-mm^2^ size per sample in treatment and denoted as the number of trichomes per mm^2^. The instantaneous water use efficiency (WUE_i_) was calculated as the ratio between photosynthetic rate (A) and transpiration rate (E), using the data obtained from a portable photosynthesis system (LI-6400 XT; LI-COR Inc. Lincoln, Nebraska, USA), recorded at 26°C temperature, and 60% RH at PAR of 1600 µmol m^−2^ s^−1^.

### Yield and quality attributes

The yield and yield components were recorded in both the plants in a replication, and the average was presented. The number of flowers produced was counted during each flowering and denoted as total number of flowers per plant. The number of fruits was counted in all seven pickings, and the total was denoted as the total number of fruits per plant. The ratio of the total number of fruits formed to the total number of flowers produced determines the fruit set per cent. Finally, fruit yield was calculated as the fresh weight of fruits in all seven pickings and expressed as kg plant^−1^. The stress tolerance efficiency was calculated based on the yield obtained in stressed and unstressed plants and expressed in percentage.

The total carotenoids and lycopene content were determined in uniformly ripened fruits as per Lichtenthaler (1987). 1 g of fruit sample was extracted using 100% acetone and centrifuged at 10,000 rpm for 15 min, until residue turned colorless, and then extracted using pure hexane. The absorbance was read at 470 nm for total carotenoids and 503 nm for lycopene. The standard curve was obtained, and the values were expressed as mg 100 g^−1^ of fresh weight. The acidity of tomato fruit samples was expressed in the percentage of citric acid and determined as mentioned in Gaikwad et al. (2020). The desired quantity of tomato fruits was crushed to obtain tomato juice, then the juice was dissolved in 10 mL of distilled water and titrated against 0.1 N sodium hydroxide solution (NaOH) containing phenolphthalein as an indicator. The 2,6-dichlorophenol indophenol method was used to estimate ascorbic acid content (AOAC, 2006). The desired quantity of fruit sample was macerated with 4% oxalic acid, and the solution was made to 50 mL, and 10 mL of filtered solution was titrated against dye. The readings are noted as the endpoint was visually seen as pink color. Ascorbic acid content was calculated using L-ascorbic acid as a standard, and the values are expressed in mg of ascorbic acid per gram of fresh weight.

### Data analysis

A factorial analysis was performed with two factors using a factorial completely randomized design (FCRD) containing four replicates. All the variables were analyzed by univariate analysis (ANOVA) to assess the significant differences between stress, foliar spray, and their interaction using SPSS for Windows, version 16.0. Chicago, SPSS Inc., software. The results were presented as mean of four replications and standard error of means (SEM). The mean comparison was performed by computing the least significant difference (LSD) test at a 5% probability level. The statistical significance was denoted by lowercase letters, indicating that the means with the same letters had no significant difference at *p* = 0.05. The graphs of all the variables were obtained using GraphPad Prism software for Windows, version 9.0.0.

## Results

**Figure 2:**
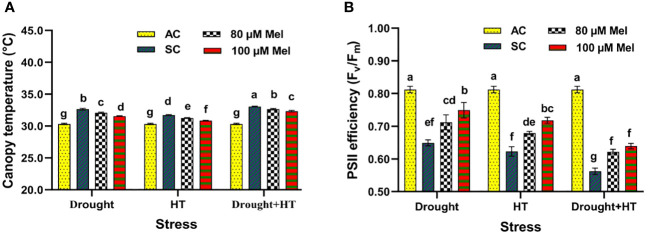
Influence of stress [drought, high-temperature (HT), and combined drought and high-temperature (drought+HT)] and foliar spray [irrigated control (AC), stress control (SC), 80 µM melatonin (80 µM Mel), and 100 µM melatonin (100 µM Mel)] on **(A)** canopy temperature. **(B)** PSII efficiency (F_v_/F_m_) on the 10th day of stress in tomato. The data represent the mean of four replications and the standard error of means (SEM). To compare the means, the least significant difference test (LSD_5%_) was computed using analysis of variance (ANOVA). Statistical significance was denoted by lowercase letters, indicating that the means with the same letters had no significant difference at *p* = 0.05.

The canopy temperature (**Figure 2A**) and PSII efficiency (**Figure 2B**) were found to have a significant effect (*p*< 0.05) in relation to the influence of stress, foliar spray, and the interaction of stress and foliar spray. Among the stresses, drought+HT stress resulted in a higher increase in canopy temperature of 9% compared with individual drought (7%) or HT (4%) stress (**Figure 2A**). Among the foliar sprays, irrespective of stress control, tomato plants exposed to drought, HT, and drought+HT stress sprayed with 80 µM melatonin showed a lower decrease in the canopy temperature of 1% than 100-µM melatonin-sprayed plants. Specifically, drought+100 µM melatonin, HT+100 µM melatonin, and drought+HT+100 µM melatonin decreased the canopy temperature by 3%, 3%, and 2%, respectively, compared with their respective stress controls and other treatment combinations. In contrast, PSII efficiency (F_v_/F_m_) was lower in drought+HT-stressed plants (30%) compared with individual HT (23%)- or drought (20%)-stressed plants (**Figure 2B**). Irrespective of absolute control, a foliar spray of 100 µM and 80 µM melatonin showed an increased F_v_/F_m_ ratio in drought-stressed plants by 16% and 10%, respectively, compared with HT, and drought+HT-stressed plants. The interaction of stress and foliar spray treatments showed that spraying 100 µM melatonin to drought+HT-stressed plants increased the F_v_/F_m_ ratio by 13%, which was lower than the increase observed in drought+100 µM melatonin (16%), and HT+100 µM melatonin (15%) sprayed plants compared with their respective stress control.

**Figure 3:**
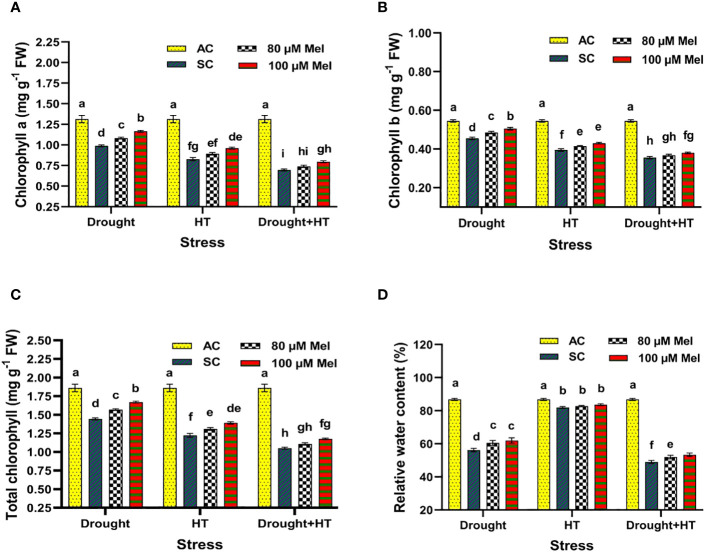
Influence of stress [drought, high-temperature (HT), and combined drought and high-temperature (drought+HT)] and foliar spray [irrigated control (AC), stress control (SC), 80 µM melatonin (80 µM Mel), and 100 µM melatonin (100 µM Mel)] on **(A)** chlorophyll a, **(B)** chlorophyll b, **(C)** total chlorophyll, and **(D)** relative water content on the 10th day of stress in tomato. The data represent the mean of four replications and the standard error of means (SEM). To compare the means, the least significant difference test (LSD_5%_) was computed using analysis of variance (ANOVA). Statistical significance was denoted by lowercase letters, indicating that the means with the same letters had no significant difference at *p* = 0.05.

The influence of stress, foliar spray, and their interaction between the stress and foliar spray showed a significant effect (*p*< 0.05) for chlorophyll a (**Figure 3A**), chlorophyll b (**Figure 3B**), total chlorophyll (**Figure 3C**), and relative water content (**Figure 3D**). Among the different stresses, drought-stressed plants showed a lesser decrease in chlorophyll contents (23%) than HT (34%) or drought+HT-stressed (43%) plants (**Figures 3A-C**). Among the foliar sprays, absolute control exhibited an increased chlorophyll content followed by 100 µM melatonin-sprayed tomato plants exposed to drought (15%), HT (13%), and drought+HT (11%) stress than other treatments. Application of 100 µM melatonin upon drought-stressed plants had a higher increase in total chlorophyll content by 15% than other treatment combinations. Also, among the treatment combinations, the contents of chlorophyll *a* and chlorophyll *b* noticing an increase in drought+100 µM melatonin-sprayed plants by 18%, and 11%, respectively than HT+100 µM melatonin (16% and 8%, respectively) and drought+HT+100 µM melatonin (13% and 6%, respectively)-sprayed plants (**Figures 3A-C**). On the other hand, drought+HT-stressed plants were found to have a greater reduction in relative water content (44%) than drought (35%) or HT (5%) stress alone ((**Figure 3D**). Among the foliar sprays, irrespective of drought, HT, and drought+HT stress control, less increase in relative water content was observed in 80 µM melatonin-sprayed plants when exposed to drought (7%), drought+HT (5%), and HT (1%) stress than 100 µM melatonin and absolute control. Among the treatment combinations, drought+100 µM melatonin-sprayed plants increased the relative water content to a greater extent by 10% than drought+HT+100 µM melatonin (8%)- and HT+100 µM melatonin (2%)-sprayed plants compared with respective stress control.

**Figure 4:**
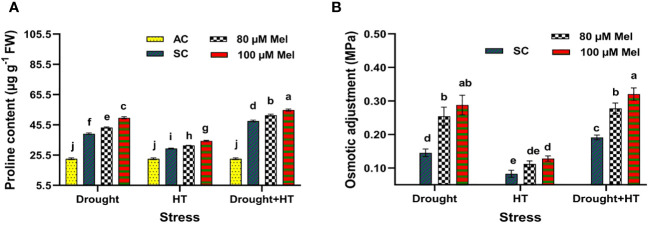
Influence of stress [drought, high-temperature (HT), and combined drought and high-temperature (drought+HT)] and foliar spray [irrigated control (AC), stress control (SC), 80 µM melatonin (80 µM Mel), and 100 µM melatonin (100 µM Mel)] on **(A)** proline and **(B)** osmotic adjustment on the 10th day of stress in tomato. The data represent the mean of four replications and the standard error of means (SEM). To compare the means, the least significant difference test (LSD_5%_) was computed using analysis of variance (ANOVA). Statistical significance was denoted by lowercase letters, indicating that the means with the same letters had no significant difference at *p* = 0.05.

The proline content (**Figure 4A**) and osmotic adjustment (**Figure 4B**) were significantly (*p*< 0.05) influenced by stress, foliar spray, and their interaction. Among the stresses, drought- and drought+HT-stressed plants exhibited an increase of 70% and 108% in proline content, respectively, than HT-stressed plants showing a 29% increase in proline content (**Figure 4A**). Among the foliar sprays, 100 µM melatonin-sprayed plants showed a pronounced increase in proline of up to 139%, 117%, and 51% in drought+HT, drought, and HT stress, respectively, than other treatments in comparison with absolute control. In comparison with drought, HT, and drought+HT stress control, drought-stressed plants sprayed with 100 µM melatonin increased the proline content by 26% than HT+100 µM melatonin (17%)-sprayed plants, drought+HT+100 µM melatonin (14%)-sprayed plants, and other treatment combinations. Similarly, the plants exposed to drought and drought+HT stress exhibited higher osmotic adjustment of 0.15 MPa and 0.19 MPa, respectively, than HT stress (0.08 MPa) (**Figure 4B**). Among the foliar sprays, irrespective of absolute control and stress control, 80 µM melatonin-sprayed plants is less osmotically adjusted to HT (0.11 MPa), drought (0.25 MPa), and drought+HT (0.28 MPa) stress than 100 µM melatonin-sprayed plants. The stress and foliar spray interaction showed that drought+HT+100 µM melatonin, drought+100 µM melatonin, and HT+100 µM melatonin-sprayed plants showed higher osmotic adjustment of approximately 0.32 MPa, 0.29 MPa, and 0.13 MPa, respectively, than other treatment combinations.

**Figure 5:**
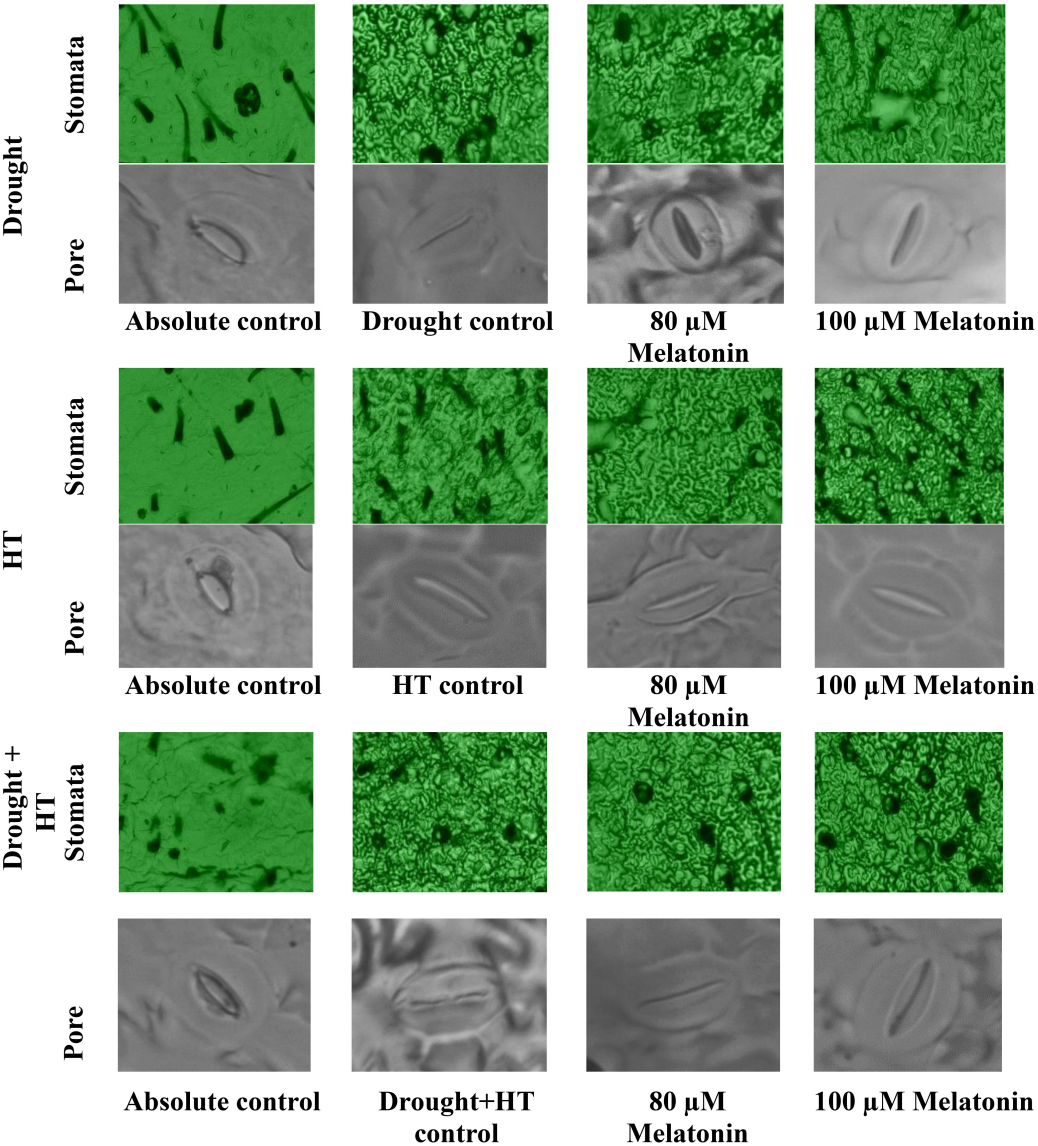
Influence of stress [drought, high-temperature (HT), and combined drought and high-temperature (drought+HT)], and foliar spray [irrigated control (AC), stress control (SC), 80 µM melatonin (80 µM Mel), and 100 µM melatonin (100 µM Mel)] on stomatal anatomy in tomato on the 10th day of stress.

**Figure 6:**
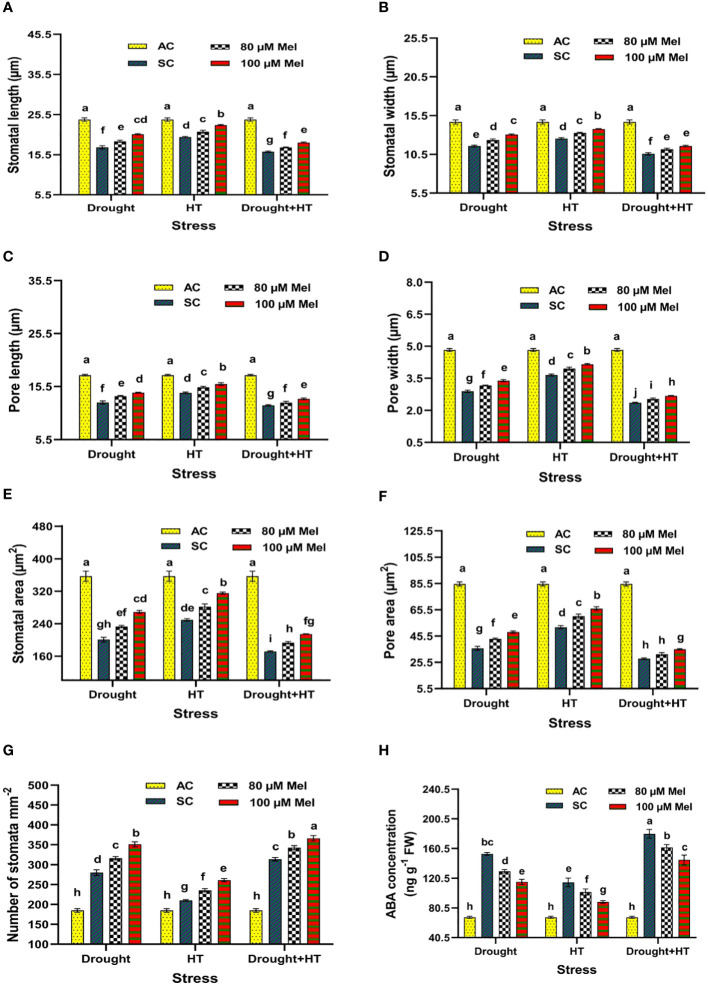
Influence of stress [drought, high-temperature (HT), and combined drought and high-temperature (drought+HT)] and foliar spray [irrigated control (AC), stress control (SC), 80 µM melatonin (80 µM Mel), and 100 µM melatonin (100 µM Mel)] on **(A)** stomatal length, **(B)** stomatal width, **(C)** pore length, **(D)** pore width, **(E)** stomatal area, **(F)** pore area, **(G)** number of stomata, and **(H)** ABA concentration on the 10th day of stress in tomato. The data represent the mean of four replications and standard error of means (SEM). To compare the means, the least significant difference test (LSD_5%_) was computed using analysis of variance (ANOVA). Statistical significance was denoted by lowercase letters, indicating that the means with the same letters had no significant difference at *p* = 0.05.

The stomatal anatomy (**Figure 5**), stomatal characteristics (**Figures 6A-G**), and ABA concentration (**Figure 6H**) exhibited significant differences (*p*< 0.05) in response to the influence of stress, foliar spray, and their interaction. Among the stresses, drought+HT stress showed decreased stomatal length (33%), stomatal width (27%), pore length (32%), pore width (51%), stomatal area (52%), and pore area (67%) to a greater extent than drought and HT stress alone (**Figures 6A-F**). Also, drought+HT stress also led to an increase in number of stomata (69%) than drought (51%) and HT (13%) stress (**Figure 6G**). Among the foliar sprays, irrespective of stress control, both 100 µM melatonin- and 80 µM melatonin-sprayed plants positively influenced the stomatal characteristics when exposed to drought, drought+HT, and HT stress. Plants sprayed with 100 µM melatonin recorded an increased stomatal length, stomatal width, stomatal area, pore length, pore width, and pore area in drought (19%, 13%, 34%, 15%, 17%, and 34%, respectively), HT (15%, 10%, 26%, 12%, 14%, and 27%, respectively), and drought+HT (14%, 9%, 24%, 10%, 13%, and 25%, respectively) stress than their respective stress control and other treatment combinations (**Figures 6A-F**). Also, among the treatment combinations, drought+100 µM melatonin-, HT+100 µM melatonin-, and drought+HT+100 µM melatonin-sprayed plants showed an increase in number of stomata by 26%, 24%, and 16% over their respective stress control, respectively (**Figure 6G**). Similarly, the tomato plants exposed to drought and drought+HT stress exhibited a onefold increase in ABA concentrations than HT stress, showing a 69% increase (**Figure 6H**). Among the foliar sprays given to drought-, HT-, and drought+HT-stressed plants, a less increase in ABA concentration was observed in 100-µM melatonin-sprayed plants by 70%, 30%, and 113%, respectively, than other treatments compared with absolute control. The stress and foliar spray interaction showed that drought+100 µM melatonin-, HT+100 µM melatonin-, and drought+HT+100 µM melatonin-sprayed plants showed a significant decrease in ABA concentration by 25%, 22%, and 19%, respectively, compared with their respective stress control and other treatment combinations.

**Figure 7:**
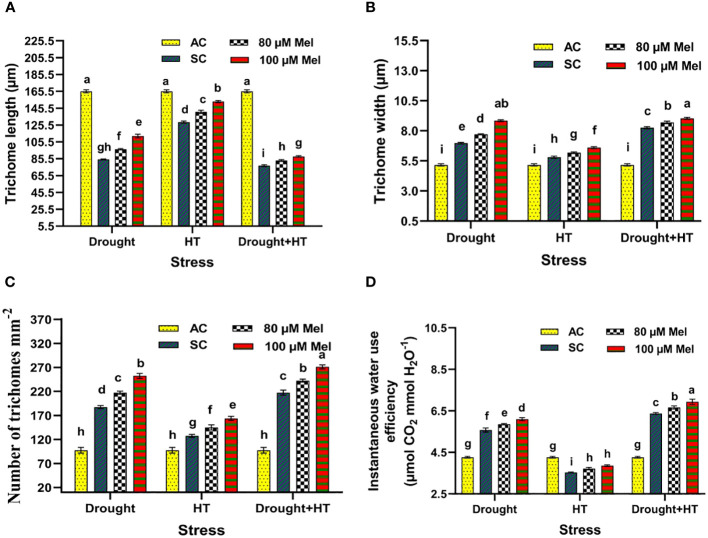
Influence of stress [drought, high-temperature (HT), and combined drought and high-temperature (drought+HT)] and foliar spray [irrigated control (AC), stress control (SC), 80 µM melatonin (80 µM Mel), and 100 µM melatonin (100 µM Mel)] on **(A)** trichome length, **(B)** trichome width, **(C)** number of trichomes, and **(D)** instantaneous water use efficiency in tomato on the 10th day of stress. The data represent the mean of four replications and the standard error of means (SEM). To compare the means, the least significant difference test (LSD_5%_) was computed using analysis of variance (ANOVA). Statistical significance was denoted by lowercase letters, indicating that the means with the same letters had no significant difference at *p* = 0.05.

**Figure 8:**
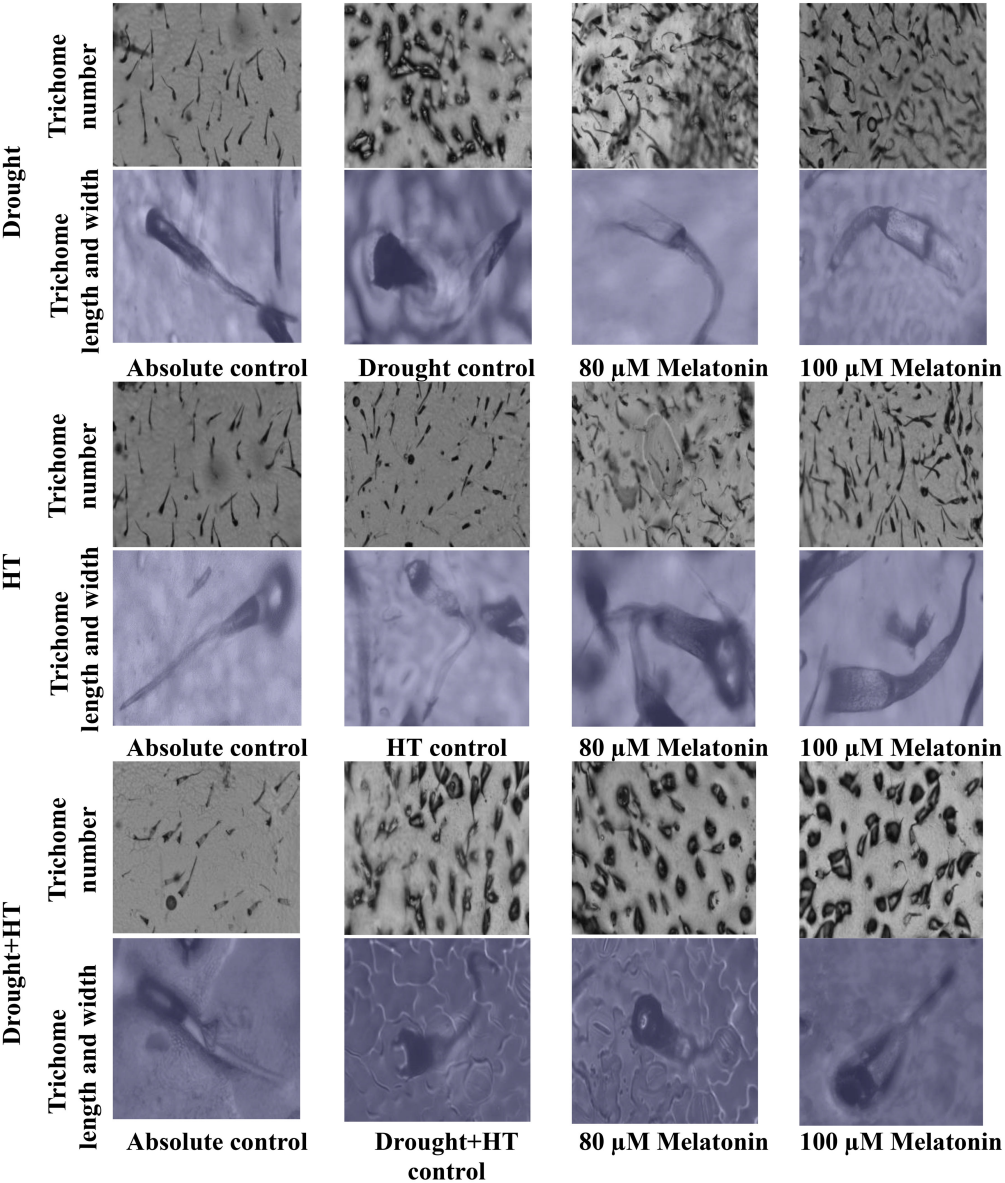
Influence of stress [drought, high-temperature (HT), and combined drought and high-temperature (drought+HT)] and foliar spray [irrigated control (AC), stress control (SC), 80 µM melatonin (80 µM Mel), and 100 µM melatonin (100 µM Mel)] on trichome anatomy in tomato on the 10th day of stress.

The trichome characteristics (**Figures 7A-C**), trichome anatomy (**Figure 8**), and WUE_i_ (**Figure 7D**) were found to have significant effects (*p*< 0.05) in relation to the influence of stress, foliar spray, and their interaction. Among the stresses, drought+HT-stressed plants showed a greater decrease in trichome length by 53% than drought (48%) or HT stress (22%). Also, the trichome width and number of trichomes were found to be increased in drought+HT-stressed plants by 60% and 123%, respectively, than drought or HT stress alone (**Figures 7A-C**). Among the foliar spray, irrespective of absolute control and stress control, 80 µM melatonin-sprayed plants showed less positive influence on the trichome characteristics when plants exposed to drought, HT, and drought+HT stress than 100 µM melatonin. Among the treatment combinations, drought+100 µM melatonin-, HT+100 µM melatonin-, and drought+HT+100 µM melatonin-sprayed plants showed an increased trichome length of 33%, 19%, and 14%, respectively, than other treatment combinations. In the case of trichome width and number of trichomes, drought+100 µM melatonin-sprayed plants showed an increase of approximately 27% and 35%, respectively, than drought control and other treatment combinations. On the other hand, WUE_i_ was higher in drought+HT-stressed plants (50%) compared with drought (31%)- or HT (17%)-stressed plants alone (**Figure 7D**). Among the foliar spray, decreased WUE_i_ was observed in absolute control. The interaction of stress and foliar spray combinations showed that 100 µM melatonin spray increased the WUE_i_ in drought- and HT-stressed plants by 9% than their respective stress control.

**Figure 9:**
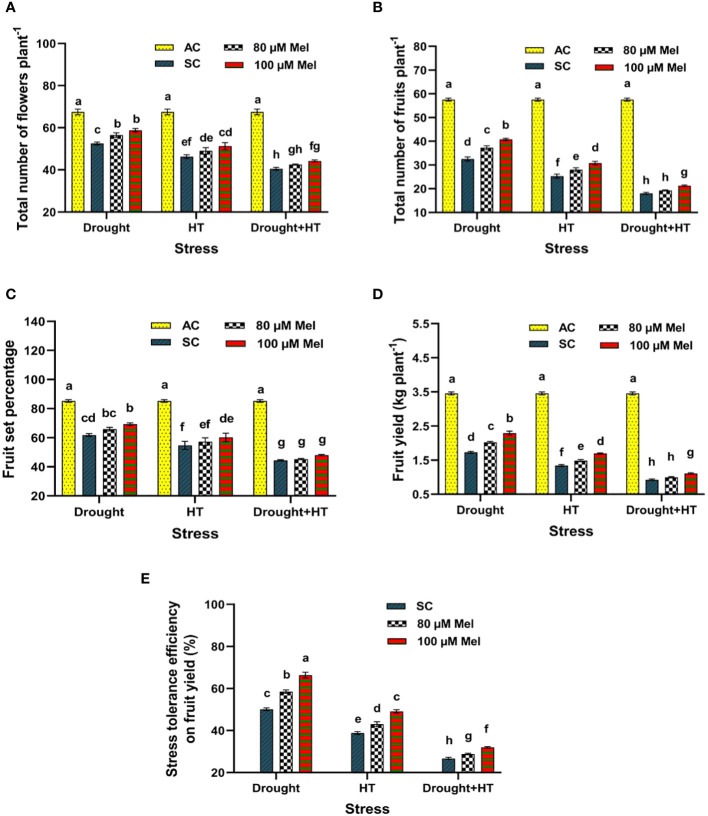
Influence of stress [drought, high-temperature (HT), and combined drought and high-temperature (drought+HT)] and foliar spray [irrigated control (AC), stress control (SC), 80 µM melatonin (80 µM Mel), and 100 µM melatonin (100 µM Mel)] on **(A)** total number of flowers **(B)** total number of fruits, **(C)** fruit set percentage, **(D)** fruit yield, and **(E)** stress tolerance efficiency on fruit yield in tomato on the 10th day of stress. The data represent the mean of four replications and standard error of means (SEM). To compare the means, the least significant difference test (LSD_5%_) was computed using analysis of variance (ANOVA). Statistical significance was denoted by lowercase letters, indicating that the means with the same letters had no significant difference at *p* = 0.05.

The stress, foliar spray, and their interaction were significant (*p*< 0.05) for yield parameters (**Figures 9A–D**) and stress tolerance efficiency (**Figure 9E**). Among the stresses, reduced number of flowers, number of fruits, fruit set percentage, and fruit yield was observed in drought+HT-stressed plants (40%, 69%, 48%, and 73%, respectively) than individual HT-stressed (31%, 56%, 35%, and 61%, respectively) or drought-stressed plants (22%, 43%, 27%, and 50%, respectively) alone (**Figures 9A-C**). Among the foliar sprays given to drought-, HT-, and drought+HT-stressed plants, absolute control showed an increase in yield and yield parameters, *viz*., number of flowers, number of fruits, and fruit set percentage, than in other treatments. The interaction of stress and foliar spray showed that drought+100 µM melatonin-sprayed plants increased the number of flowers, number of fruits, fruit set percentage, and fruit yield by 11%, 25%, 12%, and 32%, respectively, compared with drought control and other treatment combinations. The plants exposed to drought+HT stress exhibited lower stress tolerance efficiency of 26% than individual HT (39%) or drought (50%) stresses (**Figure 9E**). Among the foliar sprays, irrespective of drought, HT, and drought+HT stress control, lower stress tolerance efficiency was observed in 80 µM melatonin-sprayed plants when exposed to drought+HT (28%), HT (43%), and drought (58%) stress than 100 µM melatonin-sprayed plants. Among the treatment combinations, the stress and foliar spray interaction showed a higher stress tolerance efficiency in drought+100 µM melatonin (66%)-, HT+100 µM melatonin (49%)-, and drought+HT+100 µM melatonin (32%)-sprayed plants than their respective stress control.

The fruit quality parameters (**Table 2**) were found to have a significant effect (*p*< 0.05) in relation to the influence of stress, foliar spray, and their interaction. Among the stresses, lycopene content and total carotenoid content were significantly decreased in drought+HT-stressed plants (38% and 39%, respectively) than HT (30% and 33%, respectively)- or drought (24% and 27%, respectively)-stressed plants. However, the titratable acidity and ascorbic acid contents were increased by 40% and 41%, respectively, in drought-stressed plants than HT- or drought+HT-stressed plants. Among the foliar sprays, irrespective of absolute control, 100 µM melatonin-sprayed plants showed less decrease in lycopene content and total carotenoid content when tomato plants are exposed to drought (15% and 15%, respectively)-, HT (23% and 26%, respectively)-, and drought+HT (33% and 34%, respectively)-stressed plants. However, the foliar spray of 100 µM melatonin also showed higher increase in titratable acidity (58%, 35%, and 22%, respectively) and ascorbic acid content (60%, 78%, and 97%, respectively) in drought-, HT-, and drought+HT-stressed plants compared with absolute control. The stress and foliar spray interaction showed that drought-stressed plants sprayed with 100 µM melatonin increased the lycopene, total carotenoid content, titratable acidity, and ascorbic acid content by 12%, 11%, 12%, and 13%, respectively, compared with drought control and other treatment combinations.

**Table 2:** Influence of stress [drought, high-temperature (HT), and combined drought and high-temperature (drought+HT)] and foliar spray [irrigated control (AC), stress control (SC), 80 µM melatonin (80 µM Mel), and 100 µM melatonin (100 µM Mel)] on lycopene content, total carotenoid content, titratable acidity, and ascorbic acid content under stress in tomato.

Parameters	Treatments	Drought	HT	Drought + HT
Lycopene content (mg100 g^−1^ FW)	Absolute control	15.42 ± 0.23 ^a^	15.42 ± 0.23^a^	15.42 ± 0.23 ^a^
	Stress control	11.60 ± 0.13 ^d^	10.70 ± 0.34^e^	9.42 ± 0.17 ^g^
	80 µM melatonin	12.45 ± 0.11 ^c^	11.43 ± 0.23^d^	9.98 ± 0.11 ^fg^
	100 µM melatonin	13.04 ± 0.08 ^b^	11.79 ± 0.21^d^	10.33 ± 0.09 ^ef^
Total carotenoid content (mg100 g^−1^ FW)	Absolute control	26.53 ± 0.53 ^a^	26.53 ± 0.53^a^	26.53 ± 0.53 ^a^
	Stress control	20.24 ± 0.26 ^d^	17.72 ± 0.11^ef^	16.14 ± 0.23 ^g^
	80 µM melatonin	21.27 ± 0.26 ^c^	18.47 ± 0.13^e^	16.79 ± 0.31 ^fg^
	100 µM melatonin	22.51 ± 0.24 ^b^	19.47 ± 0.16^d^	17.37 ± 0.37 ^f^
Titratable acidity (%)	Absolute control	0.44 ± 0.01 ^f^	0.44 ± 0.01^f^	0.44 ± 0.01 ^f^
	Stress control	0.62 ± 0.01 ^b^	0.54 ± 0.01^d^	0.49 ± 0.01 ^e^
	80 µM melatonin	0.67 ± 0.02 ^a^	0.57 ± 0.02^c^	0.52 ± 0.01 ^de^
	100 µM melatonin	0.69 ± 0.01 ^a^	0.59 ± 0.01^bc^	0.53 ± 0.01 ^d^
Ascorbic acid content (mg 100 g^−1^ FW)	Absolute control	10.70 ± 0.22 ^f^	10.70 ± 0.22^f^	10.70 ± 0.22 ^f^
	Stress control	15.19 ± 0.32 ^e^	17.13 ± 0.21^d^	19.14 ± 0.33 ^c^
	80 µM melatonin	16.95 ± 0.31 ^d^	18.54 ± 0.23^c^	20.11 ± 0.18 ^b^
	100 µM melatonin	17.10 ± 0.29 ^d^	19.10 ± 0.29^c^	21.11 ± 0.18 ^a^

The data represent the mean of four replications and the standard error of means (SEM). To compare the means, the least significant difference test (LSD_5%_) was computed using analysis of variance (ANOVA). Statistical significance was denoted by lowercase letters, indicating that the means with the same letters had no significant difference at p = 0.05.

## Discussion

Crop production is significantly impacted by drought and high-temperature (HT) stress, resulting in substantial economic losses (Prasad et al., 2021). Zhang et al. (2014) reported that a combination of drought and high-temperature (drought+HT) stress is more detrimental than drought or HT stress alone. To mitigate the stress, melatonin, a biostimulator, is widely used for stimulating plant growth and development under various abiotic stress conditions, which is identified as one of the promising approaches in crop management aspects (Jahan et al., 2021). In our research, tomato plants were imposed to drought, HT, and drought+HT stress at the flowering stage to evaluate the response of melatonin on the physiological and anatomical traits on the 7th day after foliar spray, including yield and quality traits at the maturity stage.

The canopy temperature serves as a significant indicator of how plants respond to rising temperature (Thakur et al., 2022). Our research demonstrated that plants exposed to drought and drought+HT stress led to an increased canopy temperature compared with HT-stressed plants. Therefore, melatonin spray, attributed to an increase in stomatal conductance, thereby increasing the transpiration rate upon heat dissipation and help in canopy cooling, which resulted in decreased canopy temperature, which is considered as a promising characteristic for identifying stress tolerance ability in plants, as demonstrated in previous studies (Hassan et al., 2022). Increased canopy temperature and the inhibition of chlorophyll biosynthetic enzymes are primary factors contributing to the decline in photosystem II (PSII) efficiency under HT stress. Additionally, drought stress induces chlorophyll degradation and reduces leaf water content (Fahad et al., 2017). Our study indicated that plants exposed to drought, HT, and drought+HT stress exhibited decreased F_v_/F_m_, chlorophyll a, chlorophyll b, and total chlorophyll content. Moreover, relative water content, an indicator of plant water status, was significantly affected to greater extent in drought+HT-stressed plants than individual stresses. However, melatonin spray maintains a balance between chlorophyll synthesis and degradation, protects chlorophyll biosynthesis enzymes, and prevents water loss. As a result, melatonin-sprayed plants showed an increase in F_v_/F_m_, relative water content, and chlorophyll content compared with plants without melatonin spray. Similar findings have been reported in studies conducted on chrysanthemum (Luo et al., 2023) and strawberry (Manafi et al., 2021).

Osmotic adjustment evaluates the stress tolerance ability in plants, and it relies on the relative water content and osmotic potential of stressed and unstressed plants, as explained by Wu et al. (2022). Our research on melatonin showed more negative osmotic potential, enabling plants to maintain water balance under drought, HT, and drought+HT stress conditions. This finding shed light on how melatonin balances the relative water content. Stress-imposed plants enhance their osmotic adjustment by accumulating osmolytes, proline being a key osmolyte (Langaroudi et al., 2023). In our study, we also observed elevated proline levels in melatonin-sprayed plants subjected to drought, HT, and drought+HT stresses, evidencing its role in osmotic adjustment, similar to the previous study of Niu et al. (2019).

Abiotic stresses, such as drought, HT, and drought+HT, have differential effects on stomatal size. In these cases, drought and drought+HT stress cause stomatal closure, resulting in decreased stomatal width and pore width due to turgor loss (Jensen, 1981). Melatonin showed a positive influence on stomatal characteristics, although it did not have a direct impact on ABA levels. However, melatonin alleviated the negative effects of drought, HT, and drought+HT stress by reducing ABA concentration, possibly due to its interaction with other hormone signaling pathways (Jensen et al., 2023). Trichomes play a vital role in reducing water loss and resisting water flow in stressed plants by maintaining relative water content (Wang et al., 2021). Among the stresses, drought and drought+HT stress resulted in increased trichome number and width but decreased trichome length. Meanwhile, melatonin spray under drought, HT, and drought+HT stress showed a slight increase in trichome length, width, and number of trichomes compared with respective stress control. Our study demonstrated that the application of melatonin improved the stomatal and trichome characteristics, thereby resulting in increased WUE through partial stomatal closure, which is essential for improving crop productivity. The result coincides with the findings of Galdon-Armero et al. (2018).

Ripoll et al. (2014) found that drought and HT stresses disrupt carbohydrate supply and transport pathways in tomato, thus negatively impacting fruit development and quality. Our research supports this, as we observed a significant reduction in number of flowers, fruit yield, and fruit quality in plants exposed to drought, HT, and drought+HT stress. The reduced number of fruits may be due to decreased conversion of flower to fruit formation. Due to the stress exposure, the reduced or degradation of food reserves alters the pollen viability and causes stigma dryness (Khattak et al., 2022). In our experiment, we could observe the reduction, which was due to floral abortion, loss of stigmatic receptivity, and flower drop. Similar findings were reported in potato (Rykaczewska, 2017) and cucurbits (Kumar and Reddy, 2021). However, applying melatonin as a foliar spray led to notable improvements through increased numbers of fruits and overall fruit yield, which may be attributed due to enhanced photosynthate translocation from the source to the sink. It also resulted in increased fruit set percentage, which may be due to higher pollen release, improved pollen viability, and better germination (Khattak et al., 2022). Furthermore, melatonin spray also resulted in increased lycopene content, carotenoid content, titratable acidity, and ascorbic acid content in tomato. Similar findings were reported in pomegranate (Lorente-Mento et al., 2021). Ultimately, our study demonstrates that melatonin application mitigates the negative effects of drought, HT, and drought+HT stress, leading to increased fruit yield, and protects the fruits against oxidative damage, resulting in improved fruit quality.

## Conclusion

Drought, HT, and drought+HT stresses lead to an increase in canopy temperature, resulting in reduced F_v_/F_m_ in tomato. Among these stresses, drought+HT stress had a more detrimental impact than HT or drought stress alone. However, a foliar spray of 100 µM melatonin effectively increased the chlorophyll content, relative water content, and proline compared with the stress control, indicating higher osmotic adjustment. This resulted in enhanced water use efficiency, fruit yield, and fruit quality. Exogenous melatonin proves to be a successful approach in mitigating the negative effects of drought, HT, and drought+HT stress by promoting osmolyte accumulation and employing an ABA-independent stomatal closure mechanism. This study on melatonin sheds light on how plants adapt to withstand stress. Future research may focus on exploring genes that upregulate and downregulate ABA and hormonal cross talks under individual and combined drought and HT stresses. Additionally, investigating the aspects of pollen germination and root architecture can provide a clear understanding of stress avoidance or tolerance mechanism, contributing to improved crop productivity.

## Data availability statement

The original contributions presented in the study are included in the article/supplementary material. Further inquiries can be directed to the corresponding authors.

## Author contributions

AM: Formal Analysis, Investigation, Methodology, Software, Writing – original draft. SA: Conceptualization, Methodology, Resources, Funding acquisition, Supervision, Writing – review & editing. KA: Methodology, Software, Writing – review & editing. MD: Methodology, Software, Writing – review & editing. MK: Resources, Writing – review & editing. RS: Writing – review & editing. SM: Writing – review & editing. SV: Resources, Writing – review & editing. SK: Conceptualization, Methodology, Writing – review & editing.
